# A New View of Diabetes

**Published:** 1908-07-04

**Authors:** R. G. Eccles

**Affiliations:** 191 Dean Street, Borough of Brooklyn, New York City


### EDITOR'S LETTER-BOX.

### "A NEW VIEW OF DIABETES."

Sir,—Please accept my thanks for your kindness in sending me a copy of your journal containing your remarks upon "A New View of Diabetes." It is very interesting to me to see how so radical a departure from the "orthodox" way of viewing that subject is looked upon by medical men, and it is particularly so when I am able to get a glimpse into the minds of medical men of Europe. There is, to me, no more delightful study than the psychology of different methods of ratiocination. The advancement of a new hypothesis, particularly if it is a novel one, brings out the peculiar characteristics of the critics as nothing else could. While, therefore, you have been amused by my strange way of looking at the subject of diabetes, I have received equal pleasure in studying the way it has affected your thought. As I view the matter, hypotheses are made to be killed unless they can show themselves invulnerable at every point. A pet hypothesis that needs protection is the worst sort of a nuisance. From this view-point, therefore, I find no trouble in viewing, not only complacently, but with decided pleasure, any attack that may be made upon my ideas. Being a firm believer in the survival of the fit, and an equally strong believer in the idea that the final fitness is best, you can understand my indifference as regards the fate of my mental progeny.

You have called me a teleologist, and, in a certain sense, your use of the word may be correct; but in the generally accepted meaning of that word is it not just a little misleading to call a radical Darwinian such? While, in a metaphoric way, I may refer to nature's adaptations as "purposeful," it is not my habit to write "nature" with a large "N" nor to consider natural selection pregnant with intent or motive. Laveran's malarial protozoon was not, by my way of thinking, purposely designed to complete one phase of its existence within the body of a human victim. That view would make me teleological, but that is the very view I refuse to accept. I do not believe in final causes.

Are you not just a little bit hard on me when you accuse me of "labouring under a confusion of thought"? It is possible that my words did not with sufficient clearness convey my meaning. Who's always do? The intent of what I said, and what I understood Sir Frederick Treves to say, answered your query as to what disease is, "in the popular sense." The average layman knows nothing about your "diatheses," attached pieces of stone, ureters, bacillus coli communis, or "fluid of the resulting hydronephrosis." He only knows the, to him, visible symptoms, and it has been his habit to interpret all of these as evils to be subdued. Consultation of Professor Adami's contribution on Inflammation to Allbutt and Rolleston's "System of Medicine," ought to clear up your interrogatories from the medical view-point. If you are a "teleologist" you may look upon pain as a penalty for sin, and so refuse to agree with Sir Frederick Treves and myself. We look upon it as a fitting survival that has been preserved and intensified because it has compelled sufferers to refrain from acts that would be fatal. Without it the earth would be depopulated.

Symptoms that are general are adaptations, if Darwinism is true. To consider them in any other light is to deny the truth of the selective power of nature. If adaptations, then they must be conservative and beneficial.

You say that I do not tell my readers what I mean by diabetes. Unfortunately this is true. You surely could not expect me to tell them what I do not know. I am not aware of any person having yet told us just what is diabetes. There has been an attempt made to distinguish between glycosuria and diabetes, but it is purely as a matter of convenience. Until the ætiology of the disease is fully made out no one will be able to say where diabetes ends and non-diabetic glycosuria begins. We may discover that no such distinction exists. Why, then, should you expect me to do the impossible?

The words "some obstruction" removed from their context, which showed the very intent of their use to be as general as possible, did really convert it into what you call a "delightful phrase," but its total delightfulness is injected into it by your way of putting it. The special signification will appear in my next and promised paper, in which the half-finished subject will be completed. Perhaps I should not have used so abstract an expression, but I cannot yet conceive of how I could otherwise have conveyed my abstract meaning. When my concrete views appear I will be delighted at having you tell me how I could have better expressed myself.

But I must now stop, as this discussion is much longer than I intended it should be, and I fear you may consider it somewhat more than pleasant badinage owing to my habit of using serious logic on all sorts of occasions. One finds it difficult to refrain from a habit, even when he fears a misconstruction of motive. Seriously, I can only feel thankful to you for the kindly and considerate way in which you have handled a subject that is so foreign to the ordinary. When one gets out of the beaten path he should never expect to be handled with "kid gloves," and for this reason I feel that you have dealt with me in a very generous manner. With the most friendly feeling I subscribe myself,

Very truly yours, R. G. Eccles, M.D.

191 Dean Street, Borough of Brooklyn, New York City, June 21, 1908.

